# Overexpression of PON1 reduces high glucose induced renal tubular epithelial cell injury by activating PPARγ signaling pathway to alleviate diabetes nephropathy

**DOI:** 10.20945/2359-4292-2024-0377

**Published:** 2025-08-18

**Authors:** Min Wang, Xiaona Yu, Chunmei Liu, Yuan Liu

**Affiliations:** 1 Blood Purifying Center, Qingdao Central Hospital, University of Health and Rehabilitation Sciences, Qingdao, Shandong 266042, China; 2 Department of Hematologic Lymphoma, Qingdao Central Hospital, University of Health and Rehabilitation Sciences, Qingdao, Shandong 266042, China; 3 Department of Nephrology, Laizhou People’s Hospital of Shandong Province, Laizhou, Shandong 261400, China

**Keywords:** Diabetic nephropathies, Oxidative stress, Cell proliferation, Apoptosis

## Abstract

**Objective:**

To investigate the role of PON1 in diabetic nephropathy and elucidate the underlying mechanisms using a cellular model.

**Materials and methods:**

A diabetic nephropathy model was established using high glucose-induced HK-2 cells. Potential target genes and signaling pathways were identified through bioinformatics databases, and PON1 expression was manipulated to interfere with these pathways. The effects of different treatments on cell conditions were systematically evaluated.

**Results:**

PON1, the targeted gene in diabetic nephropathy, was significantly downregulated in high glucose-induced cells. The PPARγ signaling pathway was identified as closely associated with PON1, with both PPARα and PPARγ emerging as key regulators within this pathway. We observed significant increases in lactate dehydrogenase activity, malondialdehyde levels, and cell apoptosis, along with notable decreases in superoxide dismutase levels, cell viability, and cell proliferation, in the high glucose-treated group. Additionally, the expression levels of PPARα and PPARγ were also decreased. Overexpression of PON1 (pc-PON1) in the high glucose group mitigated these effects, whereas treatment with the PPARγ antagonist GW9662 reversed the protective changes induced by pc-PON1.

**Conclusion:**

Elevated PON1 levels mitigated oxidative stress and inhibited cell death, thereby promoting cell growth and alleviating diabetic nephropathy through activation of the PPARγ signaling pathway.

## INTRODUCTION

Diabetic nephropathy (DN) is a leading cause of end-stage renal disease globally (^1^) and a significant predictor of morbidity and mortality in diabetic patients (^2^), serving as a primary microvascular complication in both type 1 and type 2 diabetes (T2DM) patients (^3,4^). The hallmark pathological features of DN include glomerular capillary damage, inflammation (^5^), hyperglycemia, hypertension, and dyslipidemia (^6^), among other risk factors contributing to its onset and progression. Current interventions for DN include blood glucose management, hypertension treatment, dyslipidemia management, smoking cessation, protein restriction, and renal replacement therapy (^7^). Despite these measures, effective treatments for DN remain limited. The emerging field of gene therapy offers a promising new approach for the treatment of DN (^8^).

Research indicates that paraoxonase 1 (PON1), an enzyme primarily synthesized in the liver and detected in the circulatory system (^9^), exhibits increased activity, which serves as a favorable indicator in patients with T2DM (^10^). Elevated PON1 activity is beneficial for preventing nephropathy in noninsulin-dependent diabetes mellitus (NIDDM) and its atherosclerotic complications (^11^). Furthermore, the PON1 polymorphisms L55M and Q192R have been identified as genetic markers linked to the progression of DN (^12^), suggesting that PON1 may serve as a potential biomarker for DN. Given that oxidative stress plays a crucial role in the onset and progression of DN (^13^), animal studies indicate that increasing PON1 expression may mitigate diabetes progression via its antioxidant properties (^14^), implying that increased PON1 expression may alleviate diabetes progression through antioxidative effects.

The peroxisome proliferator-activated receptor γ (PPARγ) signaling pathway plays a critical role in various biological processes, including metabolism, inflammation, and cell differentiation (^15^). As a nuclear receptor, PPARγ regulates the expression of genes related to lipid metabolism, glucose homeostasis, and adipocyte formation (^16^). Research indicates that modulating oxidative stress, proinflammatory factors, and the PPARγ signaling pathway can mitigate cognitive dysfunction in type 2 diabetic rats (^17^). Activation of the PPARγ signaling pathway not only significantly influences the pathophysiological mechanisms of type 2 diabetes (^18^) but also inhibits DN-induced apoptosis in podocytes (^19^).

The aim of this study was to investigate the role of PON1 in diabetic nephropathy and elucidate the underlying mechanisms using a cellular model .

## METHODS AND MATERIALS

### Regents

Primary antibodies targeting Bax (2772S), Bcl-2 (15071S), PON1 (9116S), PPARα (74076S), PPARγ (2435S), and GAPDH (2118S) were acquired from Cell Signaling Technology (Beverly, USA). The kits utilized included an LDH Cytotoxicity Assay Kit (C0017), a Total Superoxide Dismutase Assay Kit with WST-8 (S0101S), a Lipid Peroxidation (MDA) Assay Kit (S0131S), a Cell Counting Kit-8 (C0038), and a TUNEL Apoptosis Assay Kit (C1088) obtained from Beyotime in Beijing, China. Furthermore, the PPARγ antagonist GW9662 was procured from MedChemExpress in Shanghai, China.

Ethics statement was not applicable. In this study, there were no clinical samples or experimental animals, only cell experiments, and the cells were obtained from commercial cell lines, which did not require ethical approval.

### Cell culture and treatments

Cultured from the American Type Culture Collection (ATCC), HK-2 cells (RRID:CVCL_0302) were subcultured once every 5 days at a ratio of 1:3, and the number of cells passaged in the experiment did not exceed 20 passages. They were cultured in dulbecco’s modified eagle medium (SH30243.01, HyClone, USA) enriched with 10% fetal bovine serum (FBS, 11011-8615, Tianhang, China) in a cell incubator (BB150, Thermo Scientific, USA) adjusted to 37 °C with 5% carbon dioxide (CO2). The intervention time for subsequent experiments was at the time of exponential cell growth. To assess the effects of elevated glucose (high glucose [HG]) levels, HK-2 cells were seeded at a concentration of 4×105 cells/well in a 12-well plate and subsequently cultivated. Next, the cells were exposed to serum-free medium comprising 20 mM glucose (HG) for 24 hours. Cells displaying active growth and a trypan blue exclusion rate exceeding 95% were selected for further experiments. Pc-PON1 and pc-negative control (pc-NC) plasmids were obtained from GenePharma in Shanghai, China. Following HG induction, HK-2 cells were transfected with 20 µmol/L pc-PON1 in the presence or absence of 10 µM GW9662 via Lipofectamine^®^ 2000 (Thermo Fisher Scientific, USA). Subsequent to this intervention, cellular analyses were conducted for operational evaluations, during which RNA and protein samples were extracted to facilitate subsequent analyses by real-time polymerase chain reaction (rtPCR) and Western blotting.

### Cell proliferation

After being seeded at a density of 1 × 10^4^, the cells were placed in a 96-well plate and cultured in DMEM under 5% CO_2_ at 37 °C. After different treatments for 24 hours, cell proliferation was evaluated via a CCK-8 kit (C0038, Beyotime, Beijing, China). At 450 nm, optical density (OD) measurements were conducted using a microplate reader. Each experiment was performed in triplicate.

### Colony formation assay

The cells were then incubated with 0.1% crystal violet for 15 minutes at 25 °C, followed by imaging. Then, acetic acid (30%) was used to dissolve the crystal violet solution for 15 minutes at 25 °C, and the OD value was detected via a spectrophotometer at 590 nm. Three repeats were conducted in each experiment.

### TUNEL double-staining assay

DNA fragmentation was detected via a TUNEL Apoptosis Assay Kit (C1088, Beyotime, Beijing, China). Initially, HK-2 cells (5×104) were placed in 96-well plates (Corning Inc., Acton, MA, USA) and allowed to adhere overnight before being subjected to various treatments for 24 hours in fresh complete medium. The cells were subsequently subjected to TUNEL at 37 °C for 1 hour, followed by rinsing with phosphate buffered saline (PBS). After the rinsing step, the HK-2 cells were subjected to diamidinyl phenyl indole (DAPI) treatment at 37 °C. Subsequently, apoptosis in the cells was observed through a fluorescence microscope (Olympus P40; Olympus, Tokyo, Japan). The trials were repeated three times.

### Measurement of the activities of malondialdehyde and superoxide dismutase

The malondialdehyde (MDA) assay kit (S0131S) and the superoxide dismutase (SOD) assay kit (S0101S) sourced from Beyotime in Beijing, China, were used to evaluate the MDA and SOD levels within the cells. Following the manufacturer’s guidelines, the OD at 525 nm was measured for each well.

### Lactate dehydrogenase assay

The examination of cellular cytotoxicity was conducted utilizing the LDH Cytotoxicity Assay Kit (C0017, Beyotime, Beijing, China) in accordance with the guidelines outlined by the manufacturer. Following the designated course of treatment and incubation, the culture supernatant was blended with the substrate mixture and incubated in a dark environment at room temperature for 30 minutes. The reaction was catalyzed by introducing the stop solution, and absorbance measurements were conducted at 490 nm utilizing a microplate reader. The results are presented as a ratio in comparison with the maximum lactate dehydrogenase (LDH) discharge activity demonstrated by the cells.

### Disease gene targets

The identified targets were those linked to DN sourced from DisGeNET (https://www.disgenet.org/home/), GeneCards (https://www.genecards.org/), and the Comparative Toxicogenomics Database (CTD; http://ctdbase.org/). The search query utilized centered on “diabetic nephropathy”, concentrating exclusively on the human species.

### Kyoto Encyclopedia of Genes and Genomes pathway enrichment analysis

The enrichment of the signaling pathway of PON1 in DN patients in comparison with healthy controls was analyzed via the Kyoto Encyclopedia of Genes and Genomes (KEGG) database. A screening condition of FDR <0.05 was applied, and the visualization of the signaling pathway enrichment outcomes was conducted using the GOplot R package.

### Quick real time polymerase chain reaction

Following the manufacturer’s guidelines, RNA was extracted from cells via the use of TRIzol reagent (Beyotime, Beijing, China). The extracted RNA was then utilized for cDNA synthesis (Beyotime, Beijing, China). Subsequently, PCR was conducted on an ABI 7900 fluorescence quantitative PCR instrument (ABI, USA). The primer sequences designed and synthesized by General Biology (Anhui) were as follows: PON1 (human): F-5’-CACGACTTAATGCTCTCCG-3’ and R-5’-CAGAGCCAGTTTCGATTCC-3’; GAPDH (human): F-5’-TCAAGATCATCAGCAATGCC-3’ and R-5’-CGATACCAAAGTTGTCATGGA-3’. The primer concentration used per sample was 0.2 µM. The initial denaturation stage lasted 10 minutes at 95 °C, after which 40 cycles of denaturation at 95 °C for 10 seconds, annealing at 52.8 °C or 56.2 °C for 15 seconds, and extension at 72 °C for 20 seconds were performed. The temperature gradient ranged from 72 to 95 °C, increasing by increments of 1 °C per step, and the gain calibration was automatically set before the first run. Standardization of mRNA expression was achieved through normalization to GAPDH expression via a modified 2^-∆∆ CT^ calculation method.

### Western blot

The cells from the different groups were collected, washed in PBS, and lysed with lysis buffer (RIPA buffer, BR0002, Best Biological; PMSF, G2008, Servicebio; protease inhibitor, BL612A, Biosharp). Total protein was extracted and quantified via a BCA assay (P0012, Beyotime, China). The acquired protein was transferred to a polyvinylidene fluoride membrane and subsequently blocked with 5% skim milk. The primary antibodies were subsequently added to the PVDF membrane, which was incubated at 4 °C overnight. For the cell samples, the PVDF membranes were exposed to primary antibodies against Bax (20 kDa, 2772S, 1:1000), Bcl-2 (26 kDa, 15071S, 1:1000), PON1 (34 kDa, 9116S, 1:1000), PPARα (52 kDa, ab126285, 1:1000), PPARγ (58 kDa, ab178860, 1:1000), and GAPDH (37 kDa, 2118S, 1:2000) during the incubation phase. All primary antibodies were from Cell Signaling Technology (Beverly, USA), except for PPARα and PPARγ, which were from Abcam. The membranes were subsequently incubated with a secondary antibody (A0201, 1:2000, Beyotime, Beijing, China). Enhanced chemiluminescence was employed for visualization of the protein bands, with GAPDH used as the internal reference protein. Densitometric assessment of the protein levels mentioned above was conducted via ImageJ (V1.8.0, National Institutes of Health, Bethesda, Maryland, USA).

### Statistical analysis

Data analysis was carried out using SPSS Statistics (Version 20, Chicago, IL, USA). The continuous data were averaged, with both addition and subtraction procedures applied, and the standard deviation was then computed. To evaluate the differences between two groups, a *t* test was performed. For scenarios involving three or more groups, one-way analisys of variance (Anova) paired with an least significant difference was employed. A significant distinction between a pair of groups was defined as *p <* 0.05.

## RESULTS

### Effects of high glucose on oxidative stress and cellular damage in HK-2 cells

**Figure 1:**
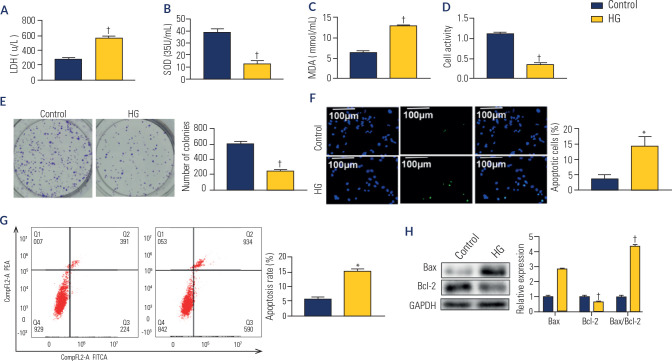
Effects of high glucose on oxidative stress and cellular damage in HK-2 cells. (**A**) The activity of lactate dehydrogenase. (**B**) The content of superoxide dismutase. (**C**) The level of activity, malondialdehyde. (**D**) Cell viability analzyed by CCK-8 kit. **(E**) Cell proliferation determined by colony formation assay. (**F**) Cell apoptosis detected by TUNEL assay (200X). (**G**) Cell apoptosis detected by flow cytometry assay. (**H**) The protein level of Bax and Bcl-2 by Western blot.

The activity of LDH and the levels of SOD and MDA were measured using commercial kits from Beyotime (Beijing, China). In HK-2 cells treated with high glucose (HG), LDH activity and MDA levels significantly increased (p < 0.001), whereas SOD levels markedly decreased (p < 0.001) (**Figures 1A** to **1C**). Cell viability and proliferation were evaluated using the CCK-8 assay and colony formation assay, respectively, whereas apoptosis was assessed via the TUNEL assay. We observed a significant reduction in cell viability and proliferation (p < 0.001; **Figures 1D** ans **1E**), along with an increase in apoptosis (p < 0.05; **Figures 1F** and **G**). This increase was accompanied by increased Bax expression and reduced Bcl-2 expression (p < 0.001; **Figure 1H**). Collectively, these findings indicate that HG treatment induced oxidative stress and cellular damage in HK-2 cells.

*p < 0.01; ^†^p < 0.001 *versus* control. HG: high glucose; DAPI: xxxx.

### Expression of PON1 in HG-induced HK-2 cells

**Figure 2:**
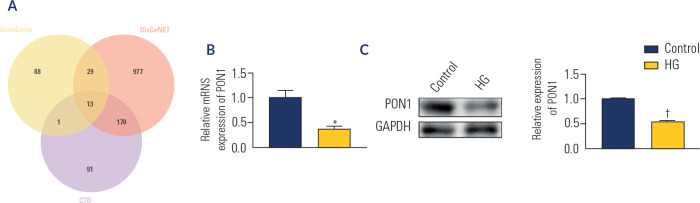
The expression of PON1 in the high glucose-induced HK-2 cells. (**A**) The screen of target gene of diabetic nephropathy by Comparative Toxicogenomics Database, DisGeNET and GeneCards database. (**B**) The expression of PON1 by real time polymerase chain reaction. (**C**) The expression of PON1 by Western blot.

Using the CTD, DisGeNET, and GeneCards databases, a comprehensive investigation was conducted to identify genes associated with DN. A total of 13 genes were identified from these databases, with PON1 emerging as the key target gene, as shown in **Figure 2A**. Subsequent analyses via real-time PCR and Western blotting were performed to evaluate PON1 expression in HG-induced HK-2 cells compared with that in the control group. The results demonstrated that in HG-induced HK-2 cells, both the mRNA (p < 0.01; **Figure 2B**) and protein (p < 0.001; **Figure 2C**) levels of PON1 were significantly reduced, indicating potential downregulation of the PON1 gene in DN.

*p < 0.01; ^†^p < 0.001 *versus* control. HG: high glucose.

### Effects of PON1 overexpression on oxidative stress and cellular damage in HG-induced HK-2 cells

**Figure 3:**
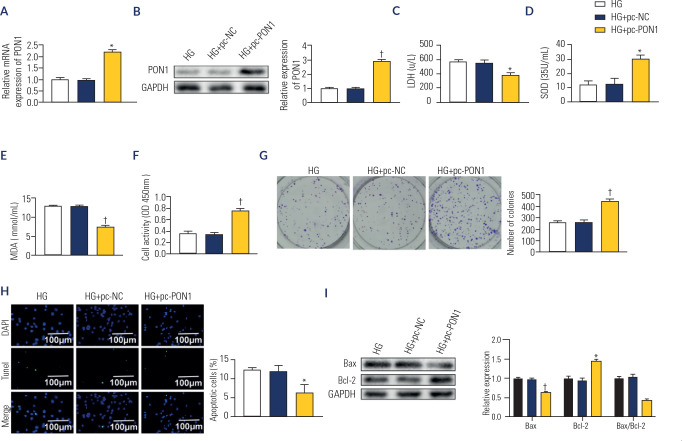
Effects of overexpression of PON1 on oxidative stress and cellular damage in HG-induced HK-2 cells. (**A**) The mRNA level of PON1 by real time polymerase chain reaction. (**B**) The protein level of PON1 by Western blot. (**C**) The activity of lactate dehydrogenase. (**D**) The content of superoxide dismutase. (**E**) The level of malondialdehyde. (F) Cell viability analyzed by CCK-8 kit. (**G**) Cell proliferation determined by colony formation assay. (**H**) Cell apoptosis detected by TUNEL assay (200X). (**I**) The protein level of Bax and Bcl-2 by Western blot.

Following transfection with pc-PON1, significant upregulation of PON1 expression was observed in HG-induced HK-2 cells, as evidenced by real-time PCR and Western blot analyses (p < 0.01; **Figures 3A** to **3B**), confirming the successful overexpression of PON1 in the *in vitro* DN model. Compared with the HG+pc-NC group, transfection with pc-PON1 resulted in decreased LDH (p < 0.01) activity and MDA levels (p < 0.001) but increased SOD levels (p < 0.01) in HG-induced HK-2 cells (**Figures 3C** to **3E**). Additionally, cell viability and proliferation were significantly enhanced (p < 0.001; **Figures 3F** and **3G**), accompanied by reduced apoptosis (p < 0.05; **Figure 3H**). This effect was associated with decreased Bax expression (p < 0.001) and increased Bcl-2 levels (p < 0.001) in HG-induced HK-2 cells transfected with pc-PON1 (**Figure 3I**). Collectively, these findings indicate that PON1 overexpression effectively mitigates HG-induced oxidative stress and cellular damage in HK-2 cells.

*p < 0.01; ^†^p < 0.001 *versus* HG+pc-negative control. HG: high glucose.

### Effects of PON1 overexpression on the PPARγ signaling pathway in high glucose-induced HK-2 cells

**Figure 4:**
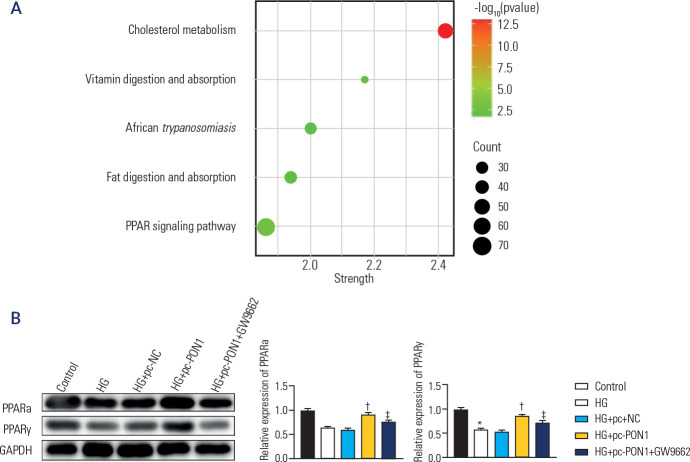
Effects of overexpression of PON1 on the PPARγ signaling pathway in high glucose-induced HK-2 cells. (**A**) Convergence of PON1-associated signaling pathways with diabetic nephropathy-involved signaling pathways. (**B**) The expression of PPARα and PPARγ by Western blot.

Through KEGG pathway enrichment analysis, the PPARγ signaling pathway emerged as the most significant pathway associated with PON1 (**Figure 4A**). We subsequently examined the expression levels of two key proteins within this pathway, PPARα and PPARγ, via Western blot analysis. As shown in **Figure 4B**, HG-induced HK-2 cells presented significantly reduced levels of both PPARα and PPARγ (p < 0.001). However, transfection with pc-PON1 partially restored their expression levels (p < 0.001), whereas treatment with GW9662, a PPARγ antagonist, further decreased these levels (p < 0.01). These findings suggest that PON1 overexpression can activate the PPARγ signaling pathway in HG-treated HK-2 cells.

*p < 0.001 *versus* control, ^†^p < 0.001 *versus* HG+pc-negative control, ^‡^p < 0.01 *versus* high glucose +pc-PON1. HG: high glucose.

### Overexpression of PON1 alleviated oxidative stress and cellular damage in high glucose-induced HK-2 cells by activating the PPARγ signaling pathway

**Figure 5:**
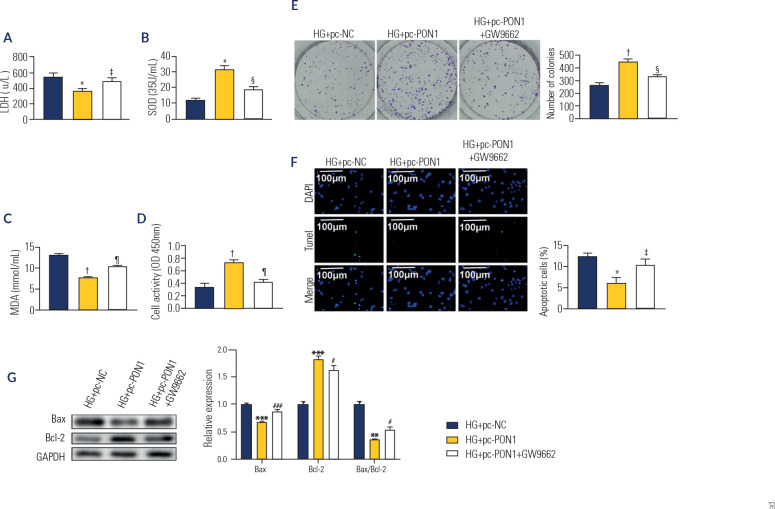
Overexpression of PON1 alleviated oxidative stress and cellular damage in high glucose-induced HK-2 cells by activating the PPARγ signaling pathway. (**A**) The activity of lactate dehydrogenase. (**B**) The content of superoxide dismutase. (**C**) The level of malondialdehyde. (**D**) Cell viability analzyed by CCK-8 kit. (E) Cell proliferation determined by colony formation assay. (**F**) Cell apoptosis detected by TUNEL assay (200X). (**G**) The protein level of Bax and Bcl-2 by Western blot.

Further investigations were conducted to elucidate the role of the PPARγ signaling pathway in mediating the effects of PON1 overexpression on DN. As shown in **Figures 5A to 5C**, compared with those in the HG+pc-NC group, the levels of LDH and MDA (p < 0.05) and the SOD content (p < 0.01) were significantly lower in the HG+pc-PON1 group. These changes were reversed by GW9662 treatment (p < 0.05). Additionally, cell viability and proliferation were markedly enhanced (p < 0.01), whereas apoptosis was reduced (p < 0.05) in the HG+pc-PON1 group, as evidenced by decreased Bax expression (p < 0.001) and increased Bcl-2 levels (p < 0.001). However, these alterations were reversed by GW9662 (p < 0.05) (**Figures 5D** to **5G**). Collectively, these findings suggest that upregulation of the PON1 gene can alleviate oxidative stress and cellular damage in HG-induced HK-2 cells via activation of the PPARγ signaling pathway.

*p < 0.01, ^†^p < 0.001 *versus* HG+pc-negative control, ^‡^p < 0.05, ^§^p < 0.01, ^¶^p < 0.001 *versus* HG+pc-PON1. HG: high glucose; NC: negative control.

## DISCUSSION

DN, characterized by cellular damage in renal cells induced by oxidative stress, is a serious complication of diabetes mellitus (^20^). Our results demonstrated that exposure of HK-2 cells to HG led to increased LDH activity and MDA levels, indicative of cellular damage and lipid peroxidation, respectively. Moreover, there was a notable decrease in SOD content, indicating impaired antioxidant defense mechanisms in response to HG-induced oxidative stress. These findings are consistent with previous studies showing similar changes in oxidative stress markers in renal cells exposed to hyperglycemic conditions (^21,22^). Moreover, we observed a significant reduction in cell viability and proliferation, along with an increase in apoptosis, in HG-treated HK-2 cells. The dysregulation of apoptosis-related proteins, characterized by increased Bax levels and decreased Bcl-2 expression, further substantiates the induction of cellular damage and apoptosis in response to high glucose exposure. These findings are consistent with previous studies demonstrating the detrimental effects of hyperglycemia on cell survival and apoptosis in renal cells (^23^).

A substantial body of research has consistently shown that PON1, which has reduced activity in both type 1 diabetes mellitus (T1DM) and T2DM patients, plays a critical role in glucose metabolism and homeostasis and is functionally involved in beta-cell insulin secretion (^24^). Through bioinformatics analysis, this study identified PON1 as a key target gene associated with DN and revealed that in HG-induced HK-2 cells, PON1 expression was markedly downregulated, suggesting a potential role of PON1 downregulation in the pathogenesis of DN. This downregulation of the PON1 gene under high-glucose conditions aligns with previous research linking reduced PON1 expression to increased oxidative stress and inflammation in diabetic conditions (^25^). To investigate the therapeutic potential of PON1 in DN, we conducted overexpression experiments in HK-2 cells exposed to high glucose. Our results demonstrated that the upregulation of the PON1 gene led to a significant reduction in LDH activity and MDA levels, whereas the SOD content increased. These changes indicate the restoration of redox balance and the mitigation of oxidative stress-induced damage. Consistent with previous studies, our findings support the antioxidant and anti-inflammatory properties of PON1 across various disease states, including diabetes and cardiovascular diseases (^26,27^). Additionally, PON1 overexpression resulted in increased cell viability and proliferation, along with reduced apoptosis, in the HG group. This effect was further supported by the modulation of apoptosis-related proteins, characterized by decreased Bax expression and increased Bcl-2 levels, reinforcing the protective role of PON1 against the cellular damage and apoptosis induced by elevated glucose levels. These results align with studies demonstrating the cytoprotective effects of PON1 under oxidative stress conditions in different cell types (^28^). Studies in PON1-deficient mice have demonstrated that PON1 deficiency results in significantly elevated lipid peroxide concentrations and markers of oxidative stress and decreased glycolysis, Krebs cycle activity, and urea cycle function. Additionally, pathways involved in triglyceride and phospholipid synthesis are markedly upregulated, whereas the pyrimidine cycle significantly increases orotate levels (^29^). These metabolic alterations collectively impact diabetes pathogenesis, suggesting that PON1 may influence diabetes through a more systemic mechanism. Consequently, further research is warranted to explore the potential of targeting PON1 for diabetes treatment.

Previous studies have revealed several potential PPARα binding sites in the PON1 gene promoter. However, the hypolipidemic drug fibrates did not cause changes in PON1 gene expression after activating PPARα. Nevertheless, rosiglitazone, a PPARγ agonist that can improve insulin sensitivity and glycemic control in patients with type 2 diabetes, was found to increase PON1 activity, although there was no significant change in the serum PON1 concentration (^30^). Additionally, antioxidant polyphenols obtained from some plants can increase PON1 expression by activating PPARγ (^31^), suggesting a strong correlation between PPARγ and PON1 in diabetes. This investigation explored the involvement of the PPARγ signaling pathway in mediating the protective effects of PON1 in the HG group and revealed that the upregulation of the PON1 gene activated the PPARγ signaling cascade, as evidenced by the restoration of PPARα and PPARγ levels in HK-2 cells under high-glucose conditions. These findings suggest that PON1 may exert its protective functions by modulating the PPARγ signaling pathway, a critical mechanism regulating oxidative stress, inflammation, and cellular viability in various disease contexts (^32,33^). Further research is warranted to investigate the therapeutic potential of PON1 and its downstream signaling pathways in managing DN.

In summary, this study examined the impact of elevated glucose on oxidative stress and cellular injury in HK-2 cells, a commonly used proximal tubular cell line. Additionally, the role of PON1 in mitigating these effects through activation of the PPARγ signaling pathway was explored. Our study elucidates the essential role of PON1 in mitigating oxidative stress and cellular damage in HK-2 cells induced by high glucose, which is achieved through activation of the PPARγ signaling cascade. These results not only increase our understanding of the molecular mechanisms underlying DN but also identify PON1 as a potential therapeutic target for reducing renal injury in diabetic patients.
